# Processing and transcriptome expansion at the mRNA 3′ end in health and disease: finding the right end

**DOI:** 10.1007/s00424-016-1828-3

**Published:** 2016-05-25

**Authors:** Anton Ogorodnikov, Yulia Kargapolova, Sven Danckwardt

**Affiliations:** Institute for Clinical Chemistry and Laboratory Medicine, University Medical Center Mainz, Langenbeckstr 1, 55131 Mainz, Germany; Center for Thrombosis and Hemostasis (CTH), University Medical Center Mainz, Langenbeckstr 1, 55131 Mainz, Germany; German Center for Cardiovascular Research (DZHK), Langenbeckstr 1, 55131 Mainz, Germany

**Keywords:** Alternative cleavage and polyadenylation (APA), Disease, Post-transcriptional gene regulation, RNA 3′ end maturation, Transcriptome diversity, Cancer

## Abstract

The human transcriptome is highly dynamic, with each cell type, tissue, and organ system expressing an ensemble of transcript isoforms that give rise to considerable diversity. Apart from alternative splicing affecting the “body” of the transcripts, extensive transcriptome diversification occurs at the 3′ end. Transcripts differing at the 3′ end can have profound physiological effects by encoding proteins with distinct functions or regulatory properties or by affecting the mRNA fate via the inclusion or exclusion of regulatory elements (such as miRNA or protein binding sites). Importantly, the dynamic regulation at the 3′ end is associated with various (patho)physiological processes, including the immune regulation but also tumorigenesis. Here, we recapitulate the mechanisms of constitutive mRNA 3′ end processing and review the current understanding of the dynamically regulated diversity at the transcriptome 3′ end. We illustrate the medical importance by presenting examples that are associated with perturbations of this process and indicate resulting implications for molecular diagnostics as well as potentially arising novel therapeutic strategies.

## Introduction

The mRNA and protein isoforms produced by alternative processing of primary RNA transcripts differ in structure, function, localization, or other properties [13, 120, 183]. Alternative splicing affects more than half of all human genes and represents a primary driver of the evolution of phenotypic complexity in mammals [15, 80, 95]. Across individuals, changes in normal isoform structure can have phenotypic consequences and have been associated with disease [52]. With the advent of next-generation RNA sequencing technologies, it became apparent that not only the “body” of transcripts but also the mRNA 3′ end is affected by enormous diversity. Up to 70 % of the transcriptome undergoes alternative mRNA 3′ end processing [92]. This for example affects numerous genes during the stress response [187] or after T and B cell activation [152]. The medical significance is highlighted by complex disorders that are associated with alternative cleavage and polyadenylation (APA), i.e., in the susceptibility to systemic lupus erythematosus [62] or more globally in tumorigenesis [119, 121]. Yet, up to now, the functional role of widespread APA in disease processes is still enigmatic.

In the following, we will briefly present the mechanistic key features of 3′ end formation since several reviews cover the basics of 3′ end formation in greater detail [28, 43, 60, 88, 143, 180, 185, 198]. We will illustrate the medical perspective of 3′ end processing and show how disease can be caused by perturbations of canonical mRNA processing in *cis* and *trans*. We will then present how alternative 3′ end processing contributes to the complexity of the transcriptome and thereby affects important cellular functions in physiological as well as pathophysiological conditions. Finally and most intriguingly, we will discuss to what extent alternative cleavage and polyadenylation represent the driver or passenger in the pathogenesis of human disorders.

## General principles of canonical 3′ end processing—the eukaryotic mRNA 3′ end cleavage and polyadenylation machinery

**Fig. 1:**
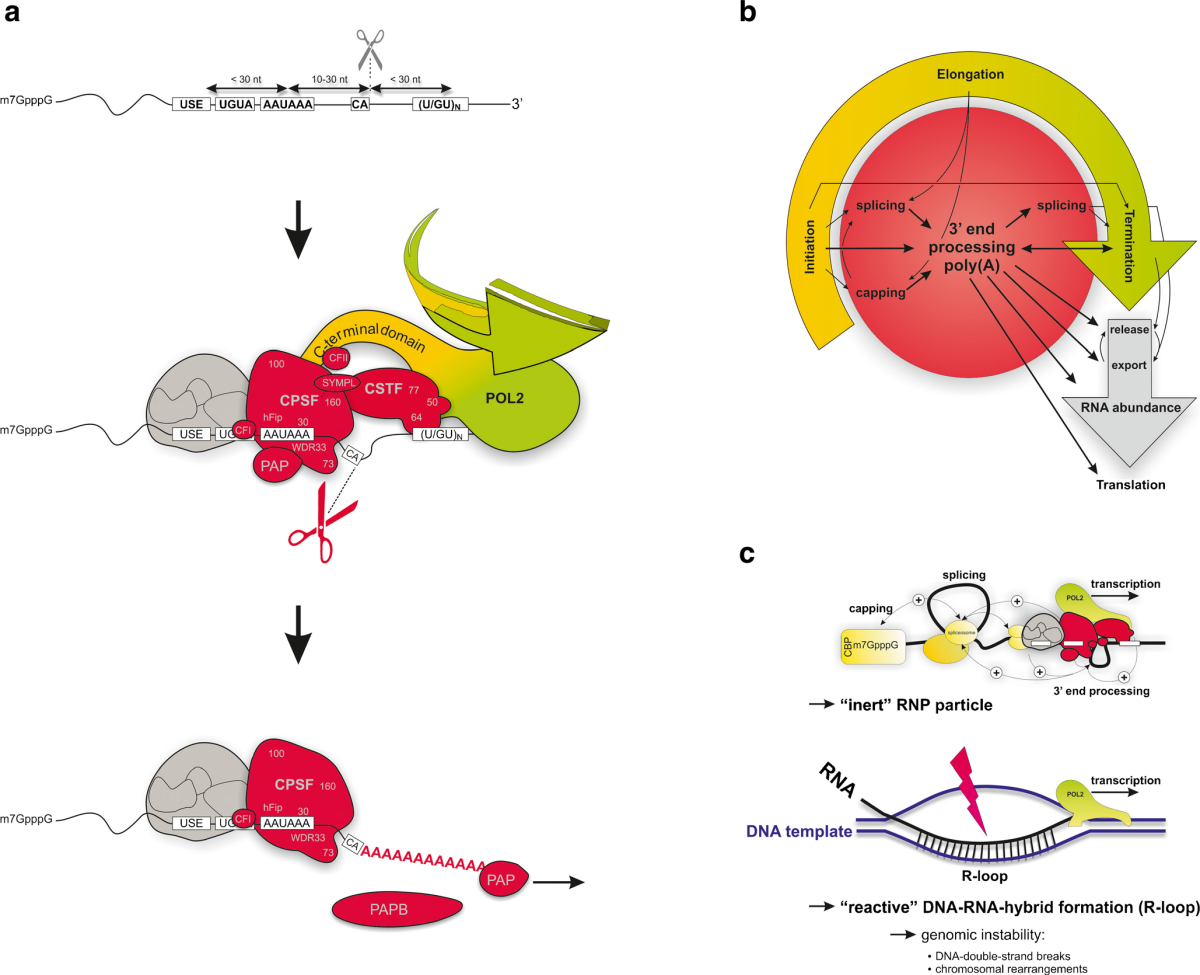
Sequence elements and protein components involved in the formation of poly(A) tails. **a** The specificity and efficiency of cleavage and polyadenylation are determined by the binding of multi-protein complexes to specific elements at the 3′ end of the pre-mRNA. Most pre-mRNAs contain two core elements. The canonical polyadenylation signal AAUAAA (or less frequently AUUAAA) upstream of the cleavage site is recognized by the multimeric cleavage and polyadenylation specificity factor (CPSF) consisting of at least six subunits (CPSF 160, CPSF 100, CPSF 73, CPSF 30, hFip1, and WDR33). This RNA-protein interaction determines the site of cleavage 10–30 nt downstream, preferentially immediately 3′ of a CA dinucleotide. The second canonical sequence element is characterized by a high density of G/U or U residues which is located up to 30 nt downstream of the cleavage site. This downstream sequence element (DSE) is bound by the 64-kDa subunit of the hetero-trimeric cleavage stimulating factor (*CSTF*) that promotes the efficiency of 3′ end processing. Furthermore, multimers of a UGUA motif are localized at variable distances upstream of the cleavage site to recruit the heterodimeric cleavage factor CFIm [19, 176]. Finally, accessory sequences can function as upstream sequence elements (USE) [17, 32, 36, 66, 67, 75, 98, 128, 129, 132, 193] to facilitate 3′ end processing by recruiting canonical 3′ end factors directly [128, 129] or by serving as an additional anchor for the (canonical) 3′ end processing machinery [36, 66]. After assembly of the basal 3′ end processing machinery, the endonucleolytic cleavage reaction is catalyzed by CPSF-73, and the cleaved mRNA is polyadenylated by nuclear poly(A)-polymerase (*PAP*). The binding of PABPN1 to the poly(A) tail is unstable, and upon nuclear export, PABPN1 is replaced by the cytosolic poly(A) binding protein (*PABPC*), which interacts with the translation initiation factor eIF4G, stimulating translation and regulating mRNA stability. Of note, in addition to histone 3′ end processing that follows a different pathway [118, 185], other “non-canonical” mechanisms of 3′ end processing exist [189]. **b** Transcription initiation, elongation, and termination (*circular arrow*) are tightly coupled to mRNA processing steps such as capping, splicing, and 3′ end processing (*inner circle*). Appropriate 3′ end processing is functionally interconnected with transcription and mRNA capping and splicing and impacts on post-transcriptional mechanisms (mRNA release, export, abundance, and translation). Loss- or gain-of-function of 3′ end processing thus critically interferes with other gene expression steps (modified after [34]). **c** Co-transcriptional mRNA processing promotes packaging of the nascent RNA transcript (formation of an “inert” RNP particle) and thus to prevent the accumulation of co-transcriptional R-loops (*lower panel*), which can lead to DNA-double-strand breaks and chromosomal rearrangements. Disruption of co-transcriptional RNA-processing can thus lead to genomic instability (modified after [34])

All eukaryotic mRNAs, except some replication dependent histone mRNAs, as well as several non-coding transcripts including miRNAs, possess poly(A)-tails at their 3′ end, which are produced by a relatively simple two-step reaction involving endonucleolytic cleavage and subsequent non-templated poly(A) tail addition (Fig. 1; reviewed [127, 142, 156, 192]). The executing molecular machinery however is complex. It involves more than 50 proteins [160], which are loaded onto more or less highly conserved sequence motifs to catalyze this step of pre-mRNA maturation. The specificity and efficiency of this process are determined by the binding of two core multi-protein complexes (CPSF and CSTF, for “cleavage and polyadenylation specificity factor” and “cleavage stimulation factor”) to sequences surrounding the poly(A) site as soon as the nascent pre-mRNA transcript emerges from the elongating RNA polymerase II (POL2). After assembling the 3′ end processing apparatus, the cleavage reaction is catalyzed by CPSF-73 [45, 112, 149] and the cleaved mRNA is polyadenylated by the nuclear poly(A)-polymerase (PAP) adding ∼50–100 A-nucleotides to the 3′ end [24]. The length of the poly(A) tail is similar in different mRNAs and is determined by an interaction between the nuclear poly(A) binding protein (PABPN1), CPSF, and PAP [91]. Upon nuclear export, PABPN1 is replaced by the cytosolic poly(A) binding protein (PABPC), which establishes contact with the translation initiation factor eIF4G, stimulating translation and regulating mRNA stability [82, 91, 150]. Thus, 3′ end polyadenylation is vital for various steps of gene regulation; it is required for nuclear export and stability of mature transcripts and for efficient translation of mRNAs [151]. Interestingly, although key aspects of mRNA 3′ end maturation have been identified decades ago, recent studies have significantly reshaped current models for the protein-RNA interactions involved in the poly(A) site (PAS) recognition [161]. This, includes the recognition of the highly conserved AAUAAA hexanucleotide motif (and less commonly AUUAAA) defining the site of 3′ end processing of almost all polyadenylated mRNAs [23, 154] (see below).

## mRNA 3′ end processing is tightly connected to transcription and splicing to ensure proper mRNA maturation and to maintain genome integrity

Messenger RNA 3′ end processing is well-orchestrated and interconnected with transcription, splicing, and translation [70, 114, 143] (Fig. 1b): As the pre-mRNA transcript emerges from POL2, extensive constitutive and alternative splicing events occur co-transcriptionally giving rise to a perplexingly high transcriptome diversity. Eventually, transcription termination pauses the elongating polymerase triggered by recognition of poly(A) signals in the nascent transcript [143, 148], and both CPSF and CSTF are transferred by the carboxy-terminal domain (CTD) of POL2 to their specific pre-mRNA binding sites to produce the mRNA 3′ end. The phosphorylation of serine and threonine residues within the CTD regulates gene expression [1]; it coordinates the recruitment of RNA processing factors (including the 5′ capping complex, the spliceosome, and 3′ processing machinery) and regulates chromatin organization by histone methylation [68].

The extensive crosstalk between the different pre-mRNA processing activities (capping, splicing, and polyadenylation) is crucial for gene expression and, importantly, genome integrity (Fig. 1c): Proteins binding to the cap structure of pre-mRNAs interact with splicing factors and promote recognition of the cap-proximal splice site. Conversely, splicing factors associating with the 3′ terminal intron interact with downstream polyadenylation factors to mutually promote 3′ end cleavage/polyadenylation and terminal intron splicing [5, 36, 65, 93, 100, 109, 122, 125, 126, 134, 175, 184]. These interactions ensure the recognition of the correct splice sites and the timely, accurate, and efficient 3′ end processing. Moreover, the extensive integration between the different co-transcriptional mechanisms protects chromosomes from potentially deleterious effects, which could arise from interaction between the nascent RNA and template DNA during transcription [101]. Finally, functional polyadenylation sites and polyadenylation factors are required for efficient transcription termination [20, 85, 143, 148] and release of the polyadenylated mRNAs for export from the nucleus [145]. The efficiency of polyadenylation can thus have significant quantitative effects on gene expression in general, and defects of mRNA 3′ end formation can profoundly affect cell viability, growth, and development.

The medical relevance of errors of 3′ end processing is exemplified by different inherited and acquired human disorders. A continuously growing number of reports document the increasing awareness of the mRNA 3′ end becoming a critical constituent for a variety of disorders (for review [34]).

In the following paragraphs, we highlight the most characteristic hallmarks of constitutive 3′ end processing and—based on a few selected examples—illustrate how alterations of sequence elements or the executing 3′ end processing machinery can result in human pathology. In the second part, we shift to regulated and alternative 3′ end formation and demonstrate its importance in various physiologically and pathophysiologically relevant processes.

## When processing gets awry

### The polyadenylation signal matters

Numerous mutations affecting *cis*-regulatory sequence elements required for mRNA 3′ end processing evidence their detrimental role in a variety of human disorders [34]. Intriguingly, a significant factor in deciphering the underlying mechanistic principles of mRNA 3′ end processing was the presence of mutations, which were initially identified in thalassemia patients. Among those patients, different mutations affecting the AAUAAA hexanucleotide of the poly(A) signal were found in the α- [69] and β-globin genes [136] (and references therein) and shown to invariably inactivate or severely inhibit gene expression. Similar mutations were observed in, e.g., the Foxp3 gene causing IPEX syndrome [10], a rare fatal disorder characterized by immune dysregulation, polyendocrinopathy, enteropathy, and X-linked inheritance. Recently, a germline variant in the TP53 polyadenylation signal has been identified in a large genome-wide association study to confer cancer susceptibility. In this gene, a mutation that changes the AATAAA into AATACA results in impaired 3′ end processing of the TP53 mRNA predisposing to prostate cancer, gliomas, and colorectal adenomas [168].

These and other examples [34] illustrate the functional importance of the highly conserved poly(A) signal and reveal the devastating consequences of some of these mutations. Although there is some sequence flexibility [9, 173] and even alternative 3′UTR architectures for effective processing [135], these findings indicate that the hexanucleotide required for recruitment of the CPSF complex represents the “Achilles heel” for disease causing loss-of-function mutations altering 3′ end processing. This is further corroborated by numerous mutations altering polyadenylation in a variety of other disorders (for review [34]).

### How about other sequence elements?

3′ end processing depends on various canonical and non-canonical (auxiliary) sequence elements (Fig. 1a). With the exception of the poly(A) hexanucleotide, they are less well conserved and consequently tolerate (“silent”) nucleotide exchanges with greater likelihood.

Nevertheless, examples, which illustrate the function of these elements, exist. This has been first demonstrated for a clinically relevant gain of function mutation of the cleavage site stimulating 3′ end processing [59]: In most cases, endonucleolytic cleavage and polyadenylation of pre-mRNAs occurs predominantly 3′ of a CA dinucleotide. However, in the prothrombin (F2) gene encoding a key blood coagulation factor, the cleavage site is composed of a CG dinucleotide, which is less efficient in promoting the cleavage reaction [26]. As a consequence, a common mutation (F2 20210*A) affecting the most 3′ nucleotide of the F2 mRNA converts the physiologically inefficient cleavage site into the mechanistically most efficient CA dinucleotide [32, 59]. This increases the cleavage site recognition resulting in an approximately twofold enhancement of F2 mRNA and protein expression. This finally causes raised F2 plasma concentrations, which disturb the finely tuned balance between pro- and anti-coagulatory activities and thereby predisposes to thrombosis [141].

Mutations affecting 3′ end processing were also identified at other positions in the F2 gene [6, 155, 165]. They increase the efficiency of 3′ end processing [33] either when located at the penultimate position of the F2 3′UTR (F2 20209*T) or further downstream 3′ of the cleavage site in the putative CSTF binding site (F2 20221*T). However, these effects are presumably gene-specific since the putative CSTF binding site in the F2 gene displays an unusually low density of uridine residues when compared to efficiently 3′ end processed mRNAs. Consequently, the introduction of (an) additional uridine-residue(s) into that region enhances 3′ end processing, supposedly by facilitating the interaction of CSTF with the pre-mRNA [32]. This illustrates a typical feature of most PASs that typically harbor a U-/GU-rich stretch up to 30 nucleotides downstream of the cleavage site to recruit the CSTF complex for efficient 3′ end cleavage and polyadenylation (Fig. 1a).

Yet deleterious effects arising from mutations have been documented, which neither directly affect the poly(A) signal or the cleavage site nor the downstream sequence elements. This, for instance, is shown for a 20 base pair duplication in MSH6, one of the four mismatch repair genes causing the LYNCH syndrome (HNPCC or hereditary nonpolyposis colorectal cancer), an autosomal-dominant genetic cancer syndrome with a high risk of colon cancer (among others) [41]. This duplication downregulates processing at an adjacent PAS, although the underlying mechanism remained unclear. Another intriguing example highlighting the mechanistic complexity of mRNA 3′ end processing is found for a complex immunodeficiency syndrome, which can be caused by a 3′UTR mutation in the p14 gene. In this case, the mutation creates a splicing defective 5′ splice site resulting in a U1 snRNP-mediated suppression of an adjacent PAS [96]. Interestingly, this principle already points to a mechanistic aspect that will be relevant in the regulation of alternative cleavage and polyadenylation and the therapeutic manipulation of 3′ end processing (see below).

These examples suggest that aberrations in regions not directly affecting the poly(A) signal deserve special attention. Although there clearly is more sequence flexibility up- and downstream of the poly(A) signal, mutations in such regions can have devastating consequences. This aspect points to another mechanistic peculiarity of 3′ end processing that the majority of RNAs likely do not have “optimal” upstream and downstream core elements. Instead, auxiliary elements situated in this regions aid polyadenylation (for review, [37]). However, their composition and mode of action are often gene-specific thereby accommodating the needs for specificity of regulation at the mRNA 3′ end (further detailed below).

### Role of *trans*-acting factors

As indicated before, the processing apparatus is a complex machinery involving more than 50 proteins [160]. Examples illustrating the medical relevance of mutations affecting 3′ end processing factors are highlighted by occulopharyngeal muscular dystrophy (OPMD) or the hypereosinophilic syndrome (HES).

OPMD is an adult-onset disease with slowly progressing muscle weakness primarily affecting the eyelids resulting in ptosis and the pharyngeal muscles resulting in dysphagia. It is caused by short trinucleotide repeat [(GCG)_8–13_] expansions in the coding region of the nuclear poly(A)binding protein 1 (PABPN1, see above) [18]. Normally, the polyalanine stretch encoded by this trinucleotide comprises 10 alanines, which is expanded to 12–17 alanines in autosomal-dominant OPMD. This expansion results in an increase of self-association, misfolding, and filamentous nuclear aggregation of the PABPN1 protein in skeletal muscle. In vitro, the mutant protein is fully active and OPMD cells do not display a severe polyadenylation defect [21, 91]. Thus, the phenotype might be best explained by either a quantitatively minor disturbance of the protein’s function in polyadenylation (which may be difficult to detect in vitro or in transfected cells) and/or by co-sequestration of other potentially interacting proteins. Finally, PABPN1 also plays an important role in the transcription of muscle-specific genes, which could explain why other tissues are unaffected [89].

In contrast, HES represents a severe hematologic disorder with sustained overproduction of eosinophils in the bone marrow, eosinophilia, tissue infiltration, and organ damage. In this case, a DNA rearrangement involving a chromosomal deletion of 800 kb and fusion of the hFip1 and PDGFRα genes is the underlying cause of this syndrome [29]. The corresponding chimeric protein, hFip1-PDGFRα, contains the N-terminus of hFip1, an integral subunit of the CPSF complex stimulating 3′ end processing [86], and the C-terminal kinase domain of PDGFRα. The expression of the hFip1-PDGFRα fusion protein in hematopoietic cells constitutively activates the PDGFRα kinase and transforms cells. As for OPMD, the resulting phenotype might further be aggravated by an interference with canonical 3′ end processing, although this has not been explored in immediate detail. Surprisingly, these examples are currently the only two reported genomic perturbations, which affect processing factors and leading to an overt phenotype. This either reflects a negative selective pressure, an unexpectedly high structural and/or functional flexibility, or the redundancy of some of those factors.

### Regulated cleavage and polyadenylation

**Fig. 2:**
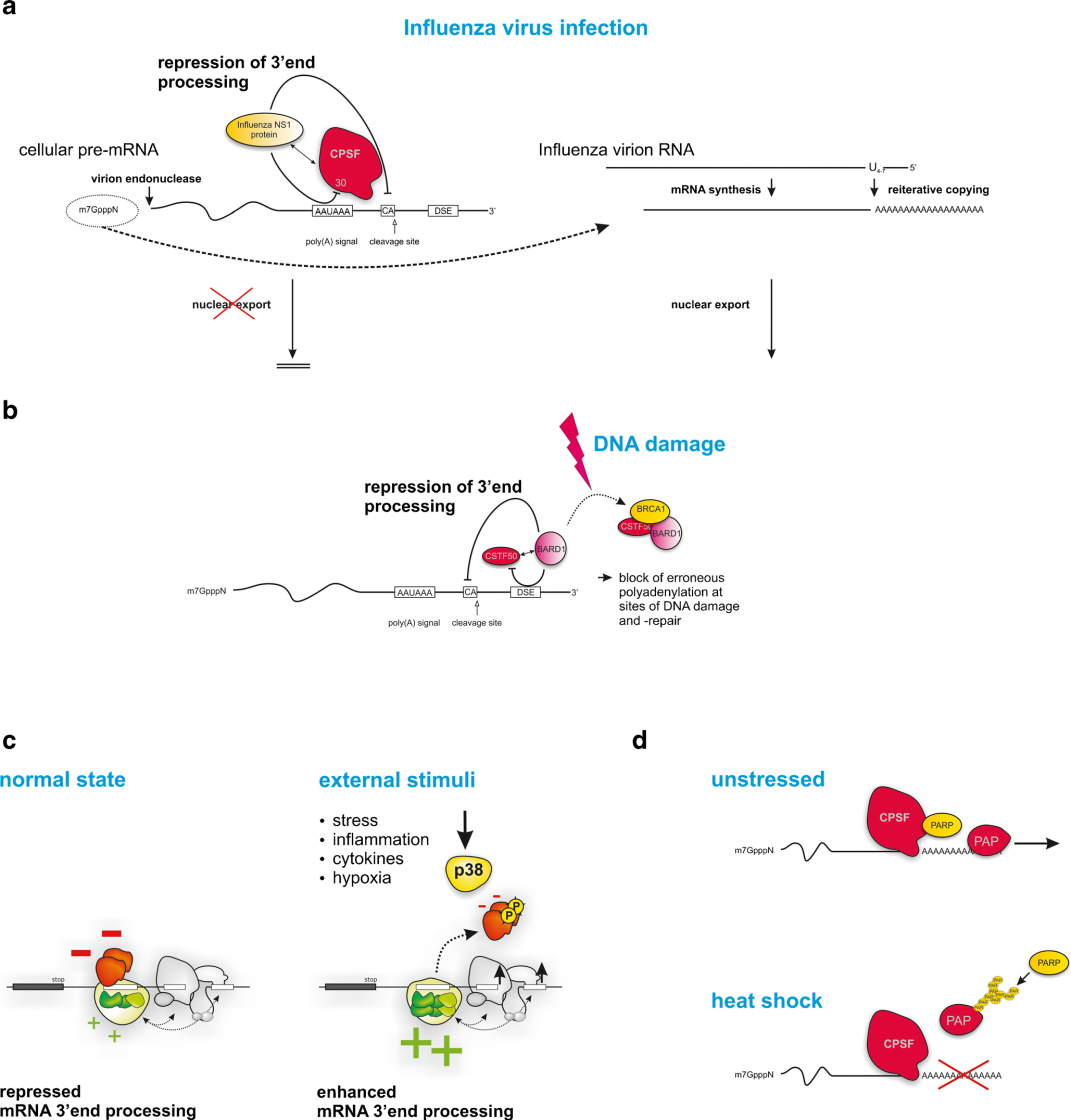
Regulated 3′ end processing in disease. **a** In influenza A virus-infected cells, the highly abundant NS1 protein interacts with the cellular 30 kDa subunit of CPSF and PABPN1 (not shown) [25]. This prevents binding of the CPSF complex to its RNA substrates and selectively inhibits 3′ end processing and nuclear export of host pre-mRNAs. In contrast, the 3′ terminal poly(A) sequence on viral mRNAs is produced by the viral transcriptase, which reiteratively copies a stretch of four to seven uridines in the virion RNA templates. In addition, an endonuclease intrinsic to the viral polymerase cleaves cellular capped RNAs to generate capped fragments that serve as primers for the viral mRNA synthesis (“cap-snatching mechanism” [144]). Thus, by interfering with the activity of an essential 3′ end processing factor, influenza has devised an efficient way to shut off cellular gene expression and to facilitate viral gene expression [133]. **b** The BRCA1-associated protein BARD1 physically interacts with CSTF-50, thereby repressing the polyadenylation machinery [90]. Both, BARD1 and CSTF-50, also interact with POL2 (not shown), and BARD1 has senses sites of DNA damage and repair. The BARD1-mediated inhibition of polyadenylation thus prevents inappropriate RNA processing during transcription at such compromised sites. Challenging cells with DNA-damaging agents results in a transient inhibition of 3′ end formation by enhanced formation of a CSTF/BARD1/BRCA1 complex. A tumor-associated germline mutation in BARD1 decreases its affinity to CSTF-50 and renders the protein inactive in polyadenylation inhibition. These findings link 3′ end RNA processing with DNA repair, and loss of wild-type BARD1 could therefore lead to defective control of gene expression as a result of inappropriate polyadenylation. **c** In the human prothrombin (F2) mRNA, the efficiency of 3′ end processing is regulated by engagement of a highly conserved USE [36]. After induction of p38 MAPK signaling the USE-RNP architecture changes [31]; inhibitory proteins binding to this element (*red*) are phosphorylated and dissociate from the USE complex while stimulatory 3′ end processing components (*green*) are more abundantly loaded onto the USE motif. This together with an induction of the canonical 3′ end processing machinery promotes 3′ end formation, resulting in higher level of F2 mRNA and protein. This process is believed to play an important role in the deregulation of blood coagulation during septicemia but also in processes such as tumor invasion. **d** The poly(A) polymerase (PAP) that catalyzes the formation of the poly(A) tail can be modified by the poly(ADP-ribose) polymerase 1 (*PARP1*). This regulates its activity both in vitro and in vivo. During heat shock, PARP1 binds to and modifies PAP leading to inhibition of polyadenylation in a PARP1-dependent manner. The inhibition reflects a reduced RNA binding affinity of PARylated PAP and decreased PAP association with non-heat shock protein-encoding genes [44]. Interestingly, this example also suggests that there must be gene-specific regulatory mechanisms that allow selective gene expression even in conditions, in which PAP as a central enzyme is posttranslationally modified to overcome the initial eliciting event (for further examples see [72])

The complexity of the macromolecular processing machinery in general and different co-factor requirements in particular offer various opportunities for a dynamic regulation of cleavage and polyadenylation ([34] and references therein). Presumably, this plays an important role in the context of physiological adaptation of gene expression but can also result in profound disease phenotypes. In analogy to the regulation of splicing, the decision how efficiently a PAS is used requires RNA sequence elements and protein regulators. Influenza A virus infections provide an interesting example of how the disturbance of protein interactions within the polyadenylation machinery can influence processing: The influenza A NS1 protein is one of the most abundant proteins synthesized in infected cells [97]. It regulates several posttranscriptional processing steps [55, 106] but also interacts with the cellular 30 kDa subunit of CPSF. This sequesters CPSF30 and thereby inhibits 3′ end cleavage and polyadenylation of the host pre-mRNAs by preventing the binding of the CPSF complex to the RNA substrate [133] (Fig. 2a). Interestingly, NS1 also targets PABPN1, which inhibits the processive synthesis of long poly(A) tails catalyzed by PAP [25]. As mRNA processing represents a prerequisite for cytoplasmic export [169], the uncleaved host pre-mRNAs are retained in the nucleus, while viral RNAs are still exported. Thus, by interfering with the activity of an essential 3′ end processing factor, influenza has devised an efficient way to shut off cellular gene expression and to facilitate viral gene expression. This and other examples [123, 124, 153] illustrate that cleavage and polyadenylation can be regulated by relative simple antagonistic molecular interactions.

However, an inhibition of 3′ end cleavage and polyadenylation can also occur as a result of endogenous cell-intrinsic mechanisms. One prominent example is the BRCA1-associated protein BARD1, which establishes a causal link between mRNA 3′ end processing and tumor suppression [90]. BARD1 senses sites of DNA damage and repair and physically interacts with CSTF-50 (Fig. 2b). Challenging cells with DNA-damaging agents transiently inhibit 3′ end formation by enhanced formation of CSTF/BARD1/BRCA1 complexes. Furthermore, a tumor-associated germline mutation in BARD1 (Gln564His) decreases its affinity for CSTF-50 and renders the protein inactive in polyadenylation inhibition. The BARD1-mediated inhibition of polyadenylation may thus prevent inappropriate RNA processing during transcription of damaged DNA loci.

Competitive protein interactions modulating the efficiency of cleavage and polyadenylation can be regulated by specific signaling pathways. This has first been demonstrated for the prothrombin (F2) pre-mRNA in which processing relies on a highly conserved upstream sequence element (USE) (Fig. 1a) [31]. In this example, stress conditions that activate p38 MAPK signaling up-modulate components of the 3′ end processing apparatus and phosphorylate the RNA-binding proteins FBP2 and FBP3 (Fig. 2c red complex). Normally, these proteins bind to the USE and inhibit 3′ end processing. Upon phosphorylation, they dissociate from the USE, making it accessible to proteins that stimulate 3′ end processing [36]. These findings have important implications: deregulated F2 expression plays a crucial role in the pathogenesis of thrombophilia but also in other conditions linking p38 MAPK activation with aberrant cellular processes such as tumorigenesis [35]. It is worth noting that the USE motif constitutes a highly conserved nonameric sequence element, which can be found in many genes including MYC (among other key players with a role in tumorigenesis [36]). Thus, this regulatory principle might account for a plethora of gene functions. From these findings, regulated 3′ end processing emerged as a key mechanism of gene regulation with broad biological and medical implications. It also provides a first example how the basal 3′ end processing apparatus is “wired” to signaling pathways allowing a dynamic adaptation of the 3′ end cleavage and polyadenylation efficiency. In analogy to other mechanisms (i.e., splicing), this also exemplifies how accessory sequence elements confer specificity to this type of gene regulation.

Finally, another important example establishing the role of posttranslational modifications as a critical element for regulation of 3′ end processing is shown for the poly(A) polymerase [44]. This enzyme catalyzing the formation of the poly(A) tail can be posttranslationally modified by the poly(ADP-ribose) polymerase 1 (PARP1) leading to a poly(ADP-ribosyl)ation, which inhibits the PAP activity (Fig. 2d). The physiological importance of this mechanism is shown in the context of heat shock during which PARP1 inhibits polyadenylation of non-heat shock protein-encoding genes, while polyadenylation of *hsp* transcripts remains unaltered. Thus, a PARP1-mediated modification of PAP has evolved as an effective mechanism for a differential regulation of polyadenylation during thermal stress. Although not fully elucidated, this example also suggests that there must be gene-specific regulatory mechanisms which allow selective gene expression even in conditions, where PAP as a central enzyme is posttranslationally modified [44].

These and other examples illustrate that complex molecular mechanisms have evolved to control and regulate mRNA 3′ end processing at (a) defined PAS(s) to eventually execute specific cellular programs. Although not yet explored in further detail, analogous mechanisms might also come into play for the dynamic regulation at alternative (“competing”) PASs (next section).

## Variations at the transcriptome 3′ end—when processing gets alternative

With the emergence of RNA sequencing (RNA-Seq) technologies, it became clear that the transcriptome is enormously diversified at the 3′ end [39]. Approximately up to 70 % of the transcriptome is affected by a mechanism widely referred to as “alternative 3′ end cleavage and polyadenylation” (APA) [92]. As highlighted above, it regulates numerous genes during the stress response or after T and B cell activation, during differentiation and dedifferentiation, and in various processes linked to tumor progression (detailed below). These findings are in line with earlier observations that alternative PAS selection represents an important and evolutionary conserved regulatory mechanism for spatial (tissue specificity [53, 67, 105, 107]) and temporal control of gene expression (i.e., immunoglobulin class-switch [3, 30, 47, 48, 147, 170, 171]).

**Fig. 3:**
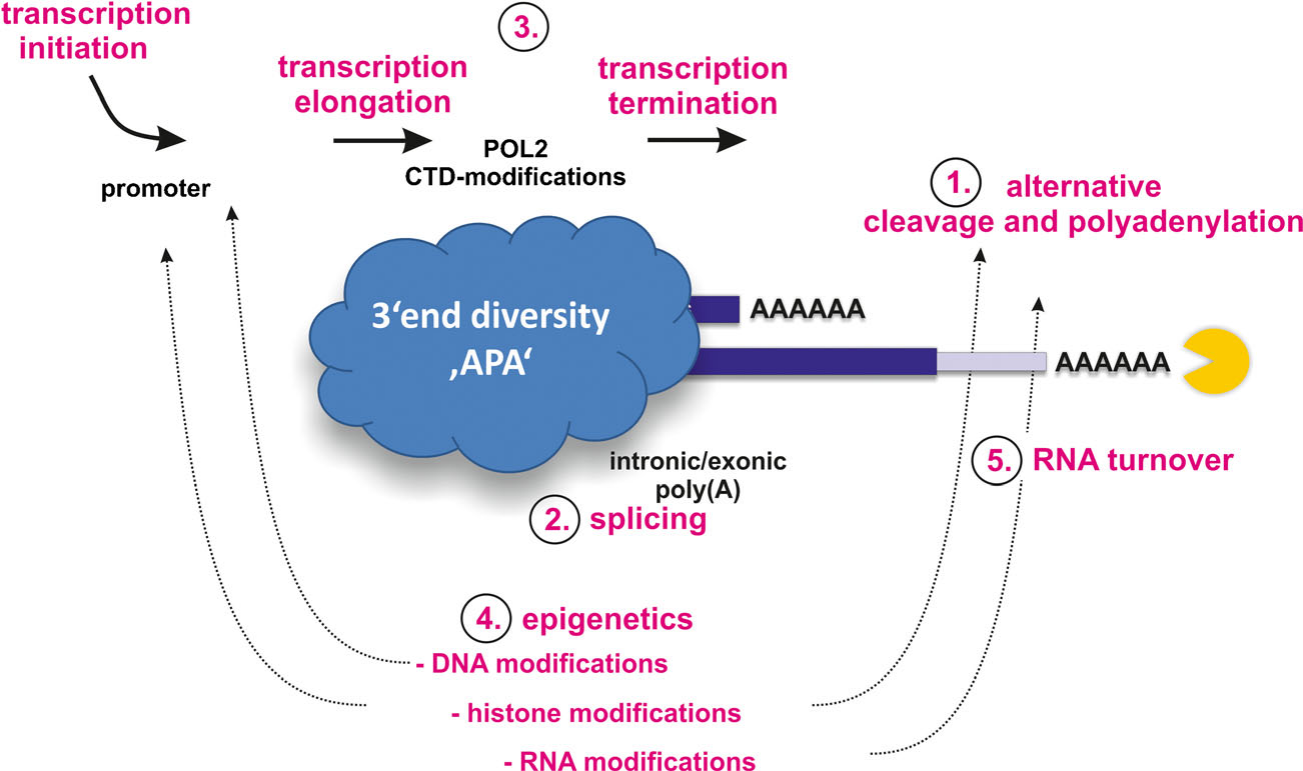
Mechanisms involved in the regulation of the transcriptome 3′ end diversity. Alternative 3′ end cleavage and polyadenylation (APA; two transcript isoforms shown) can be regulated (1) on the level of mRNA 3′ end processing (“direct/true APA”), through (2) alternative splicing via the in- or exclusion of PASs upon intron retention or exon skipping (“splicing coupled APA”) [183], by (3) transcriptional activities (transcription initiation, elongation, or termination; “kinetic coupling”), or (4) as a result of epigenetic regulation (i.e., through histone or DNA modifications; “epigenetic APA”). Ultimately, changing APA profiles can also be caused by (5) differential RNA turnover of individual mRNA isoforms, which have been cleaved and polyadenylated at alternative PAS (“faux/indirect APA”). Of note, another interesting mechanistic combination of both splicing regulation and concomitant transcriptional activities is executed via a U1 snRNP-mediated mechanism termed “telescripting” that protects pre-mRNAs from drastic premature termination by cleavage and polyadenylation [11, 83] (*APA* = alterative cleavage and polyadenylation; *CTD* = C-terminal domain; *POL2* = RNA polymerase II)

**Fig. 4:**
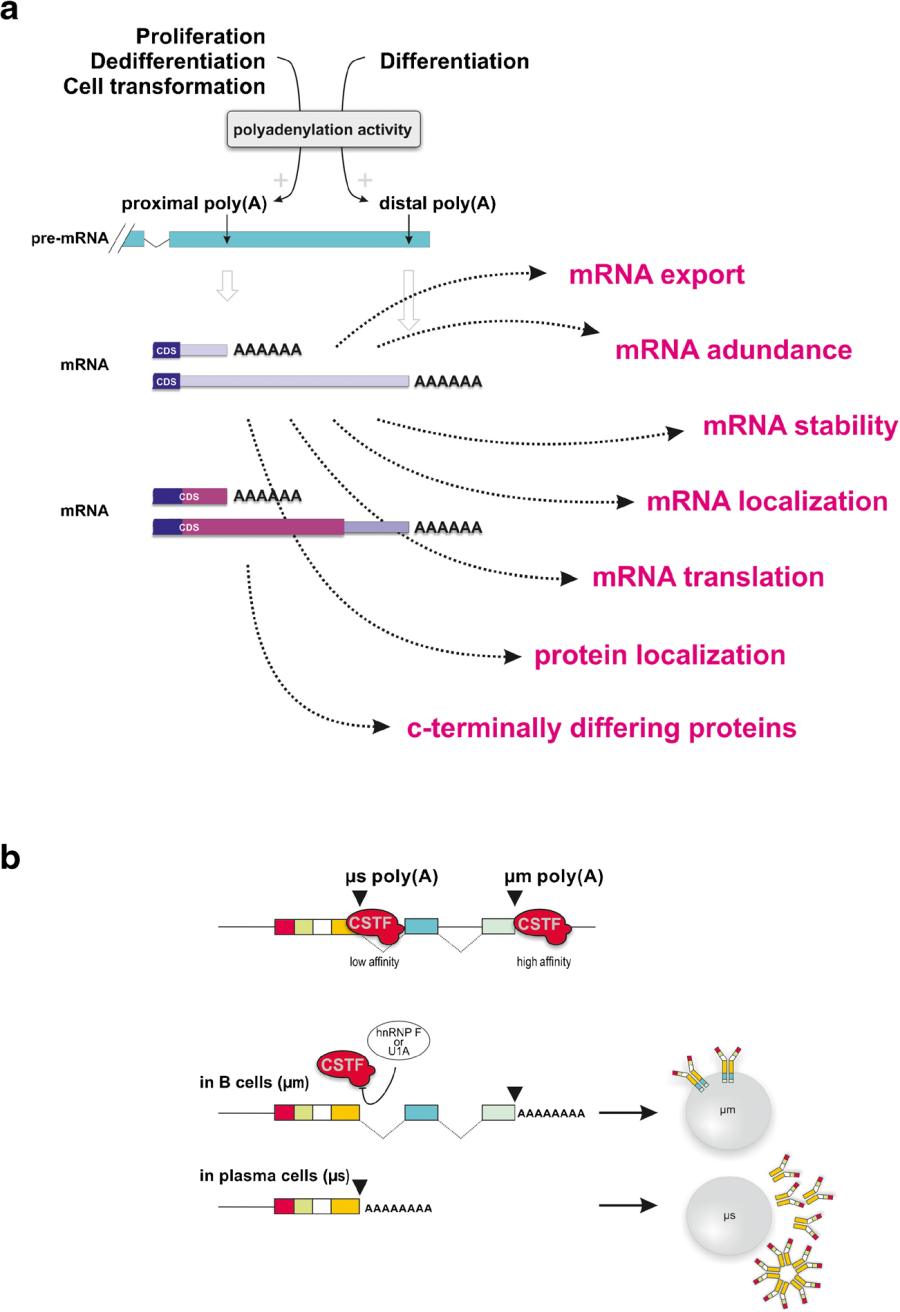
Alternative 3′ end processing modulates the temporal and spatial diversity of gene expression. **a** About half of the human pre-mRNAs contain (multiple) alternative poly(A) signals (PAS), which are mostly located as tandem arrays within the 3′-UTRs [173, 194]. A smaller set of pre-mRNAs bears alternative PAS within intronic or exonic regions. In both cases, endogenous and exogenous factors can modulate pre-mRNA PAS selection by interfering with constitutive and/or auxiliary 3′ end processing factors/subunits. This results in various polyadenylated mRNA isoforms that either code for identical (tandem terminal PASs) or C-terminally modified (internal PASs) proteins. Furthermore, alternatively 3′ end processed mRNAs can display different 3′UTR properties. This can affect various aspects of mRNA and/or protein fates (export, abundance, stability, localization, transport, and translation), i.e., via the in- or exclusion of regulatory elements such as microRNA target sites or binding sites for RNA binding proteins with important roles in Mendelian disease [22]. Very commonly, cellular states associated with enhanced proliferation, dedifferentiation, or cell transformation tend to produce shorter mRNA variants (cleavage and polyadenylation occurring at the proximal PAS), while differentiation shifts cleavage and polyadenylation towards production of longer mRNA variants (processed at the distal PAS). **b** During B cell differentiation, alternative PAS selection effects a switch of the IgM heavy chain expression from a membrane bound form (μm) to the secreted form (μs). In this example, CSTF-64 binding to the PAS of the RNA giving rise to secreted IgM (μs) is favored either by high CSTF-64 concentrations [171] or under conditions of low hnRNP F and/or low U1A concentrations [139, 177] in plasma cells (*lower lane*). In contrast, the high affinity site of the membrane bound form (μm) is used in B cells (*upper lane*) where the CSTF-64 concentration is low or when high concentrations of U1A and/or hnRNP F inhibit CSTF-64 binding to the secretory μs-specific PAS (*boxes* indicate exons, simplified representation)

The current understanding of how APA is mechanistically controlled is subject of several recent review articles [51, 63, 74, 108, 110, 159, 161, 174]. Although great steps towards a better understanding of APA have been taken, many facets are still enigmatic. Following from above, and possibly despite the fact that APA is widespread, the existence of a unique (and universal?) APA-regulating mechanism is unlikely: In brief, APA can be regulated on the level of mRNA 3′ end processing (“direct/true APA”) by various *cis*- and *trans*-acting determinants among which (1) the intrinsic strength of sequence elements, (2) the concentration or activity of polyadenylation factors, and/or (3) tissue- or stage-specific regulatory factors are the most important key players [7] (Fig. 3). Yet a significant proportion of APA events is coupled to alternative splicing; PASs located in intronic or alternatively spliced exonic regions can be activated and thus the modulation of splicing can directly influence PAS choice (“splicing coupled APA”; see below). Further, as can be inferred from the coupling of cleavage and polyadenylation with transcription (Fig. 1), transcription initiation, the kinetics of transcription itself as well as transcription termination can control PAS choice (“kinetic coupling”; see below). In addition, epigenetic regulation (i.e., through histone or DNA modifications) as well as RNA modifications can determine where and to what extent individual pre-mRNAs are eventually cleaved and polyadenylated (“epigenetic APA”) [57, 87, 102, 167, 182, 190]. Ultimately, it needs to be noted that changing APA phenotypes can also be caused by regulated RNA decay of individual mRNA isoforms, which have been constitutively cleaved and polyadenyated at alternative PAS (“faux/indirect APA”). Thus, dissecting the underlying mechanisms of regulated transcriptome 3′ end diversity is complex and has to take into account various variables that importantly can at the same time be cause and consequence of APA (Figs. 3 and 4).

Before moving on to a more universal role of APA in health and disease, we will briefly illustrate a few important physiological aspects, which deserve special attention.

## APA in differentiation and development

Recent studies based on high throughput analyses have revealed that APA is highly regulated during development ([8, 54, 76, 79, 152, 158] and references therein). Interestingly there is a correlation between the proliferation status and the global APA patterns (Fig. 4a). Proliferating cells tend to use upstream (“proximal”) PASs and produce mRNAs with shorter 3′UTRs, while quiescent/differentiated cells favor downstream (“distal”) PASs and produce mRNAs with longer 3′UTRs [152]. Similar observations have been made for mouse development and the differentiation of ECSs into neurons and other functions [16, 78, 158]. In contrast, during somatic reprogramming [79] or tumorigenesis, proximal PASs are favored leading to shorter 3′UTRs [121]. In some of those cases, mRNAs with shorter 3′UTRs tend to be more stable [71, 162] or globally elevated [40] eventually leading to higher protein output [121, 152]. Breaking it down to individual transcripts, the consequences of APA however can be complex: APA transcript isoforms of the same gene can encode different proteins and/or change the 3′UTR properties, leading to the inclusion or exclusion of mRNA stabilizing or destabilizing elements, miRNA target sites, or result in different translation efficiencies or subcellular localization (Fig. 4a, detailed below). Thus, although APA is widespread in processes such as differentiation, dedifferentiation, and development, global equations of how the overall trend of APA directionality affects the fate of the respective transcript isoforms are difficult. This is supported by studies, which failed to detect a straightforward correlation between APA and mRNA stability or protein output [50, 58, 64, 130, 152, 166]. Accordingly, deciphering the downstream functional consequences of APA in the context of cellular programs and to understand whether APA is the cause or consequence of complex biological programs represents a major challenge. Possibly APA coordinately regulates post-transcriptional regulons (“RNA operons”) driving specific cellular programs. For example, hFip1 (an integral part of the CPSF complex) has recently been shown to control embryonic stem cell-specific APA profiles to ensure the optimal expression of a specific set of genes, including critical self-renewal factors, in the cell fate specification [94]. Yet, we have just begun to decipher the resulting consequences, and further studies are needed to expand our understanding of the resulting consequences. In contrast, the impact of APA on the expression and function of individual genes is far better understood.

## Role of APA for individual genes

The historically eldest and perhaps the most thoroughly studied example illustrating the importance of APA and also shedding light on the underlying regulatory principles is the regulation of IgM heavy chain expression during B cell differentiation (Fig. 4b; [3, 47, 147]). In this mRNA, alternative PAS selection is regulated by a modulated recruitment of the CSTF 64-kDa subunit to one of two competing PASs. Upon B cell activation, this switches the IgM heavy chain expression from a membrane bound form (μm) to the secreted form (μs) by activation of an alternative upstream μs-specific PAS in plasma cells [171]. Several mechanisms are possibly contributing including additional modulators (U1A) tightly controlling and ensuring appropriate PAS selection [138, 139]. Although the underlying regulation is further complicated by being coupled to alternative splicing (“splicing coupled APA”), it establishes an important direct functional link between dynamically regulated APA and a physiological process.

A similar mechanism underlies the regulated expression of the transcription factor NF-ATc during T cell differentiation [27]. Two longer isoforms of NF-ATc mRNA are synthesized in naïve T cells, whereas a shorter isoform is expressed in effector cells. The switch is mediated by activation of a proximal PAS by up-regulation of CSTF 64 kDa subunit, which occurs upon T cell stimulation (“direct APA”). In this context, it is interesting to note that LPS stimulation increases CSTF 64 expression in macrophages, which in turn regulates APA of several mRNAs [157]. These examples illustrate that the modulated expression of individual 3′ end processing factors can directly—or in concert with auxiliary (inhibitory or stimulatory) factors—change the production of APA isoforms and thereby drive important cellular functions.

Inferring from the tight coupling of transcription with processing [115, 131], the kinetics of POL2 play an important role in PAS choice (“kinetic coupling”). In *Drosophila*, *polo* is a cell cycle gene, which uses two PAS in the 3′ UTR to produce alternative messenger RNAs that differ in their 3′ UTR length. By using a mutant *Drosophila* strain with a lower transcriptional elongation rate, it was shown that transcription kinetics can determine alternative PAS selection. Although only one gene is affected, the physiological consequences of incorrect *polo* PAS choice are detrimental; transgenic flies lacking the distal poly(A) signal cannot produce the longer transcript and die at the pupa stage due to a failure in the proliferation of the precursor cells of the abdomen [140]. Along these lines also, transcription elongation factors can direct alternative RNA processing and thereby control important cellular functions such as the immunoglobulin secretion in plasma cells [117].

Another interesting example is the brain-derived neurotrophic factor (BDNF), which is encoded by two transcripts with either short or long 3′ UTRs. The physiological significance of the two mRNA isoforms encoding the same protein has been unknown until it could be demonstrated that the short and long 3′ UTR BDNF mRNAs are involved in different cellular functions. The short 3′ UTR mRNAs are restricted to somata, whereas the long 3′ UTR mRNAs are also localized in dendrites. In a mouse mutant where the long 3′ UTR is truncated, dendritic targeting of BDNF mRNAs is impaired, resulting in low level BDNF in hippocampal dendrites, a selective impairment in long-term potentiation in dendrites, while somata of hippocampal neurons remained normal. These results provide insights into local and dendritic actions of BDNF and reveal APA for a differential regulation of subcellular functions of proteins [4] with important medical implications [103].

Further examples documenting the biological consequences of APA are represented by the regulated expression of a truncated form of glutamyl-prolyl tRNA synthetase (EPRS), which as a “gamma-interferon-*a*ctivated *i*nhibitor of *t*ranslation” (GAIT) constituent controls the translation of GAIT target transcripts such as the VEGF-A [195]. Furthermore, recent studies demonstrated APA’s potential to differentially regulate the localization of membrane proteins by a trafficking mechanism involving the CD47 3′UTR as a scaffold [12]. Altogether, these and other examples illustrate the physiological importance of regulated mRNA 3′ end processing as a mechanism controlling a wide spectrum of cellular functions.

## APA in human disease

Aberrant APA profiles are associated with a variety of human disorders ([8, 40, 119, 121, 130] and references therein). Most importantly, the strong prevalence of APA regulation in physiological processes such as differentiation and development (see above) is also reflected in situations where these processes are typically dysregulated. The most prototypical example for this is uncontrolled cellular proliferation in the course of cancer development. Accordingly, a widespread increase in the use of proximal PASs has been observed in various cancer cells [121]. In this context, the shorter mRNA isoforms showed an increased stability and typically produced more protein, in part through the loss of microRNA-mediated repression. Interestingly, switching to shorter 3′UTRs also allowed proto-oncogenes to escape from inhibition by miRNAs, thereby resulting in oncogene activation in the absence of genetic alterations. Global induction of proximal PAS usage has consistently been observed in several studies ever since ([2, 58, 104, 119, 130, 162, 191] and references therein). This phenotype however does not apply to all tumor types [58], and occasionally the correlation between cancer progression and 3′UTR shortening appears to be more complex [49, 58, 130]. Interestingly, the determination of (selected) APA profiles has recently proven to be of prognostic significance [99, 119, 186, 191]. Yet, also other disorders such as endocrine [146] or cardiovascular disease [164] are associated with a widespread regulation of APA.

Previously, PABPN1 has been identified to represent a potent modulator of APA by inhibiting processing at respective PASs [77]. In the context of OPMD (see above), these data may also imply that OPMD is associated with misregulated APA, which results in unbalanced formation of alternative mRNA 3′ ends. They also predict that OPMD symptoms in humans may be to a certain degree a result of aberrant gene expression due to a change in 3′ end formation. Yet, so far, this has not been tested directly.

Interestingly, also pathological changes occurring in the context of myotonic dystrophy may be attributable to specific alterations in 3′UTR structures and subsequent changes in RNA localization and/or protein isoforms and levels [8]. Although primarily demonstrated in mice, complementary analysis of samples of human origin strongly suggests APA to represent a (additional) molecular mechanism underlying muscular dystrophy.

Given the high prevalence of APA as a pervasive gene regulatory mechanism in various physiologically relevant processes, it is likely that its misregulation will be discovered in the context of various other pathological conditions [172]. Yet the extent to which the vast majority of all reported global alterations at the mRNA 3′ end represent driver or passengers of human disorders remains an open question. Furthermore, it is, for example, conceivable that the observed APA pattern changes could equally reflect compensatory activities reestablishing the cellular homeostasis in response to disease-triggering events.

Potentially interesting evidence for a causal relationship between APA and a resulting disorder is represented by a polymorphism in the interferon regulatory factor (IRF) 5 gene predisposing to systemic lupus erythematosus (SLE) [61, 62]. This newly identified polymorphism creates a functional polyadenylation site resulting in an increased expression of a transcript variant containing a shorter 3′UTR. Interestingly, the expression levels of transcript variants with the shorter or longer 3′UTRs appeared to be inversely correlated. Thereby, it contributes to a misregulation of interferon signaling, a critical constituent in the pathogenesis or progression of SLE. Yet, this is obviously one of the “simpler” examples in which APA (of one gene) is altered in *cis*. Accordingly, the nature and functional consequences of global APA regulation in the context of human pathologies remain subject to future investigations.

## APA in molecular diagnostics

**Fig. 5:**
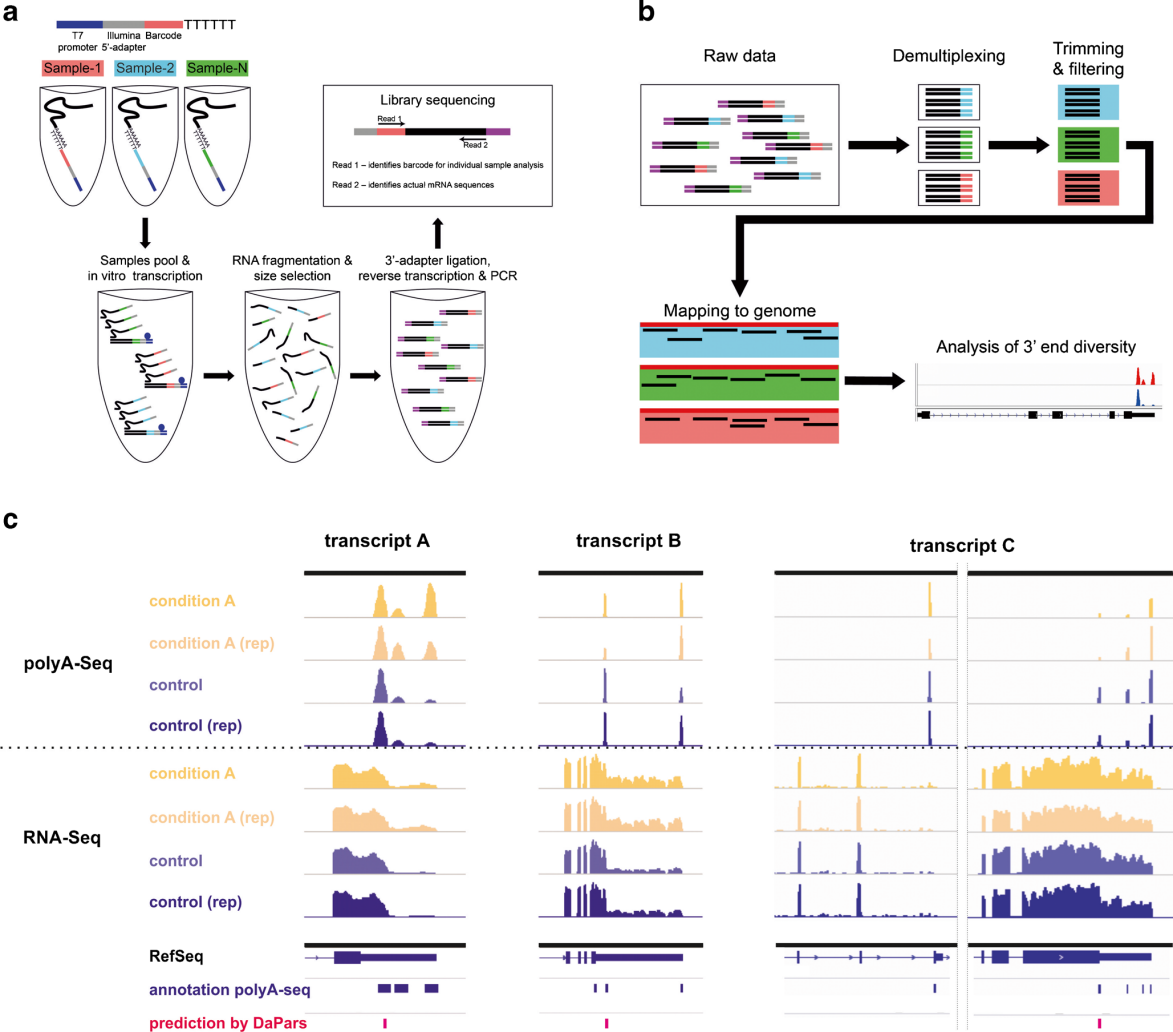
High throughput sequencing for the (multiplexed) definition of mRNA 3′ end diversity of gene expression. **a** Principle for the definition of the transcriptome 3′ end (“polyA-Seq”) via (multiplexed) high-throughput sequencing based on oligo(d)t primed, barcoded cDNA library generation. **b** Workflow showing the bioinformatical processing of the sequencing reads involving demultiplexing, filtering, and mapping for the visualization of 3′ end seq reads. **c** Visualization of the transcriptome 3′ end (three selected examples) by polyA-Seq compared to RNA-Seq and definition of the mRNA 3′ end by DaPars on the basis of RNA-Seq data [191]. Shown are differential changes of the mRNA 3′ end signature comparing two conditions (“condition A” and “control,” each in biological replicates (Danckwardt lab, unpublished)). The *lower panel* illustrates the respective RefSeq 3′UTR and the annotation of the polyA-Seq data in comparison with a differential use of PAS in DaPars

The advent of high-throughput sequencing technologies has significantly promoted the elucidation of disease mechanisms and equipped us with novel diagnostic opportunities. As for alternative splicing, identifying APA patterns can likely have wide diagnostic implications [38, 84, 181]. With the evolution of numerous protocols that rely on 3′ end sequencing technologies [42, 56, 58, 73, 76, 77, 104, 113, 116, 137, 158, 183, 188, 196], the determination of APA isoforms can meanwhile be carried out in a reliable and straightforward manner (Fig. 5). Unlike gene expression profiling based on arrays or full RNA-Seq, which can be dramatically confounded by the way of normalization, the relative proportion of APA isoforms is normally internally controlled thus resulting in relatively robust results. While contamination of the analyzed specimen by other cell populations is another inevitable and very common confounder in gene expression analysis (i.e., when revealing the signature of a tumor which is infiltrated by immune cells), APA patterns are likely to be more tissue specific [42] and were found to differ according to tissue type, developmental stage, genotype, or cancer subtype [78, 79, 92, 111, 152, 162, 183, 197]. Cataloging these (tissue) specific patterns might therefore allow us to subtract “contaminating” APA signatures from APA signatures of specific disordered tissues of interest. Thus, the determination of APA patterns may open up novel diagnostic avenues which up to this point have turned out to represent challenging aspects of “conventional” gene expression profiling. Finally, characterizing tissue-specific APA signatures per se may be of immediate diagnostic value, i.e., for tracing back and identifying the origin of a given disease lesion (for instance in cases of *cancer of unknown primary*, CUPs).

With the advent of high-throughput analyses, the bioinformatical workload has increased dramatically. In contrast to total RNA-Seq, the sequencing restricted to the transcriptome 3′ end directly uncovers the variability and perturbation occurring at the mRNA 3′ end (Fig. 5). This has several advantages. Firstly, it drastically reduces the bioinformatical workload. Furthermore, these data are typically not confounded by other variables that complicate the bioinformatical processing of the data (such as alternative splicing). Finally, restricting the sequencing to the last (approximately) 30 nucleotides of the transcriptome opens up interesting (and first and foremost cost-effective) opportunities for multiplexing—while still keeping a high coverage for a reliable analysis. In depth APA profile studies have recently revealed “aberrant” APA signatures to be associated with more aggressive tumor phenotypes in cancer patients and thereby provided the proof-of-concept that such a determination can reveal prognostic signatures [119, 191]. Yet, applying novel bioinformatical analysis (DaPars), APA patterns can also be extracted from preexisting transcriptome wide sequencing data [191]. Although this takes advantage of the fact that RNA-Seq data is already available for numerous tissue specimens, this technique has the limitation that it is primarily suited to detect alternative 3′UTR events, while APA events, which are located within the coding region, or alternatively spliced introns (internal APA) rather remain obscure. Compared to 3′ end sequencing technologies, this algorithm requires complex bioinformatical calculations and typically allows a less “intuitive” identification of the mRNA 3′ end (Fig. 5c, compare polyA-Seq with DaPars).

It remains to be observed in which disease conditions and to what extent the analysis of APA signatures could further improve diagnostic strategies and possibly allow detecting biological aberrations with higher sensitivity and specificity. Interestingly, selected APA events can confer strong prognostic power beyond common clinical and molecular variables, suggesting their potential as novel prognostic biomarkers [191]. Thus, it will be interesting to see how the determination of APA patterns may evolve as a potentially new biomarker in the future. This could advance diagnostic strategies for a more thorough understanding of underlying disease mechanisms as well as for a reliable prognostic and possibly therapeutic stratification.

Ultimately, ongoing genome sequencing activities will most likely grant us further insights into genomic variations resulting in gene-specific perturbation of APA isoforms with possible detrimental functional consequences. Unlike global aberration in *trans* (i.e., as a result of a change of the abundance of one processing factor or regulatory protein), the cause-consequence relationship in this kind of setting is substantively clearer. Further such changes may be directly accessible for specific, targeted therapeutic approaches.

## Targeting mRNA 3′ end formation as a novel therapy

We have seen that the determination of global APA patterns can have important diagnostic and even prognostic implications. The therapeutic significance of APA will depend on it being cause, consequence, or simply a coincidental epiphenomenon of the underlying disease.

However, even the latter two conditions do not necessarily preclude the possibility of regulating APA as a therapeutically meaningful approach. Various disorders are associated with drastic APA changes (see above), and it is difficult to imagine that all observed APA patterns are biologically “silent” and consequently do not affect a potential phenotype.

Although still on an experimental level, in principle, strategies to interfere with 3′ end processing are available. This encompasses both unspecific as well as target-specific strategies. For example, as shown for the regulation of splicing, antisense oligonucleotides (ASO) inhibiting U1 binding can be used to specifically promote intronic alternative polyadenylation [179]. Although the experience concerning therapeutic targeting of splicing is far more advanced, the general proof-of-concept of targeting specific polyadenylation sites for redirection of processing based on analogous approaches has been made. This includes the use of ASOs [179] and siRNAs [178] as well as modified U1 snRNP, which interacts with a target gene upstream of its PAS to regulate gene expression [14].

Yet, also other strategies might come into play as well. We have seen that APA is influenced by various other cellular processes controlling gene expression (Figs. 1 and 3) including the velocity/kinetics of POL2 [140]. Although presumably unspecific, the interference with POL2 processivity at various stages of transcription [81] may potentially regulate APA [46]. In fact, numerous anticancer drugs regulate in the one or the other way the processivity of POL2 (such as doxorubicin or camptothecin). Apart from this, the C-terminal domain of POL2 is subject to extensive posttranslational phosphorylation, which influences co-transcriptional events including splicing, transcription termination, and 3′ end processing [68]. Interestingly, although Ser 2 and Ser 5 phosphorylations of the CTD are by far the most studied posttranslational modifications, the way in which the phosphorylation pattern itself or potentially even other posttranslational modifications might affect the loading and delivery of processing factors to their ultimate destination is yet to be elucidated.

Furthermore, 3′ end processing is tightly bound to splicing, and a significant proportion of APA events per se occur concurrently with alternative splicing. Therefore, virtually all therapeutical approaches currently tested for manipulating splicing [163] may—in the one or the other instance—help reverting “disordered” APA phenotypes as well. Ultimately, we have obtained first evidence how extracellular signals influence the basal 3′ end processing machinery [31]. Generally, this and other examples connecting posttranslational modifications with the regulation of 3′ end processing as shown for PAP [44] or the modifications of POL2 CTD (see above) may lead new ways towards targeting signaling components for regulation of the transcriptome 3′ end diversity. Finally, by means of the ongoing research elucidating how epigenetic modifications can control APA switches ([102, 182, 190] and references therein), it is tempting to speculate that the manipulation of these pathways may eventually be translated into the clinical context.

## Conclusions and perspectives

The 3′UTR has emerged as a hotspot for posttranscriptional gene regulation, controlling important cellular functions such as morphogenesis, cell differentiation, metabolism, cell proliferation, and other processes by controlling mRNA translation, stability, localization, as well as 3′ end processing. More recently, APA has been found to represent an important layer of posttranscriptional gene regulation, which in turn can influence the RNA fate and/or regulate the protein output quantitatively or qualitatively, and thereby steer important cellular programs. Interestingly, we have seen distorted APA signatures being associated with a variety of disorders. Presumably, more of these patterns will be discovered in the context of various other pathological conditions in the near future.

From a medical perspective, the most intriguing question relates to the extent to which APA represents a driver or passenger of human disorders. For individual genes harboring mutations that perturb alternative cleavage and polyadenylation of their own transcripts, the cause-consequence relationship is relatively simple. However, the causal contribution of a widespread APA (de)regulation in the context of human pathologies is still unclear. The broad biological importance of APA renders the possibility of global changes of the transcriptome 3′ end (associated with human pathologies) to have any phenotypic consequences unlikely. Even without being disease eliciting directly, these changes may aggravate underlying pathologies. Yet, they could equally represent compensatory activities for reestablishing the cellular homeostasis in response to disease-triggering events.

Thus, further studies are required to decipher the functional contribution of regulated APA in the context of human pathologies in order to determine whether APA can serve as a meaningful therapeutic target. Defining key components directing APA and dissecting their functional hierarchy thus represent critical aspects that influence the conceptual tractability—apart from all practical possibilities/opportunities. Uncoupled from these challenging aspects, exiting first steps towards APA serving as a potentially novel biomarker are taken. It will be interesting to see how these encouraging findings will further translate into a clinical setting and whether they will become part of “routine” molecular diagnostics allowing prognostic and/or therapeutic stratifications in the (near) future.
